# Neuronize v2: Bridging the Gap Between Existing Proprietary Tools to Optimize Neuroscientific Workflows

**DOI:** 10.3389/fnana.2020.585793

**Published:** 2020-10-06

**Authors:** Ivan Velasco, Pablo Toharia, Ruth Benavides-Piccione, Isabel Fernaud-Espinosa, Juan P. Brito, Susana Mata, Javier DeFelipe, Luis Pastor, Sofia Bayona

**Affiliations:** ^1^Department of Computer Science, Universidad Rey Juan Carlos, Madrid, Spain; ^2^DATSI, ETSIINF, Universidad Politécnica de Madrid, Spain; ^3^Centro de Investigación Biomédica en Red sobre Enfermedades Neurodegenerativas, Madrid, Spain; ^4^Laboratorio Cajal de Circuitos Corticales, Centro de Tecnología Biomédica, Universidad Politécnica de Madrid, Madrid, Spain; ^5^Instituto Cajal (CSIC), Madrid, Spain; ^6^DLSIIS, ETSIINF, Universidad Politécnica de Madrid, Madrid, Spain; ^7^Center for Computational Simulation, Universidad Politécnica de Madrid, Madrid, Spain

**Keywords:** neuron morphology, interoperability, neuronal tracing, spine meshes, data sharing, 3D morphological reconstruction, pyramidal structure

## Abstract

Knowledge about neuron morphology is key to understanding brain structure and function. There are a variety of software tools that are used to segment and trace the neuron morphology. However, these tools usually utilize proprietary formats. This causes interoperability problems since the information extracted with one tool cannot be used in other tools. This article aims to improve neuronal reconstruction workflows by facilitating the interoperability between two of the most commonly used software tools—Neurolucida (NL) and Imaris (Filament Tracer). The new functionality has been included in an existing tool—Neuronize—giving rise to its second version. Neuronize v2 makes it possible to automatically use the data extracted with Imaris Filament Tracer to generate a tracing with dendritic spine information that can be read directly by NL. It also includes some other new features, such as the ability to unify and/or correct inaccurately-formed meshes (i.e., dendritic spines) and to calculate new metrics. This tool greatly facilitates the process of neuronal reconstruction, bridging the gap between existing proprietary tools to optimize neuroscientific workflows.

## Introduction

In recent years, the emergence of novel methods that provide new insights into the organization of the brain has produced a wealth of data that needs to be analyzed, shifting the bottleneck from the acquisition to the analysis of data. In particular, analyzing neuron morphology is essential to better understand cell functioning (Segev and Rall, 1998; Spruston, 2008), including dendritic spines (for simplicity, spines), which are of great relevance for the study of brain processing (Yuste, 2010; Heck and Benavides-Piccione, 2015). Several laboratories have made significant contributions in recent times to gathering data on and analyzing neuron morphology. These studies have contributed significantly to better understand the diversity and regional specialization of the cortical organization (e.g., Huttenlocher and Dabholkar, 1997; Cline, 1999; Preuss, 2001; Elston and DeFelipe, 2002; Jacobs and Scheibel, 2002; Elston, 2003; Luebke, 2017).

There are several software tools that aim to facilitate the reconstructions of the neurons, employing different approaches—either extracting tracings from stacks of images, as is the case for Snake (Wang et al., 2011), APP2 (Xiao and Peng, 2013), flNeuronTool (Ming et al., 2013), SmartTracing (Chen et al., 2015), NeuTube (Feng et al., 2015), Rivulet (Liu et al., 2016), TreeMap (Zhou et al., 2016), NeuroGPS-Tree (Quan et al., 2016), Neuron tracer (Wang et al., 2017) and ShuTu (Jin et al., 2019); or processing neuronal tracings as occurs with Lasserre (Lasserre et al., 2012), Filament editor (Dercksen et al., 2014), NeuTube (Feng et al., 2015) and NeuroMorphoVis (Abdellah et al., 2018). Some tools use the neuronal tracings to create 3D meshes; for example, NeuroTessMesh (Garcia-Cantero et al., 2017), NeuroMorphoVis (Abdellah et al., 2018), Neuronize (Brito et al., 2013), and Lasserre (Lasserre et al., 2012). These tools facilitate the automatic tracing of neuronal structures and the correction of tracing errors to make reconstructions more accurate and efficient. In particular, the first version of the tool Neuronize was conceived to overcome the problems caused by the unrealistic (or absent) image of the somata and low-quality unconnected 3D meshes, which are typically generated with other existing neuronal tools based on computer-aided tracings. Some of the most frequently used available reconstruction software suites are Neurolucida (NL; MicroBrightfield, VT, USA) and Imaris (BitplaneAG, Zurich, Switzerland). However, these tools are based on proprietary formats. This usually results in little or no interoperability between the tools, as users cannot reuse the data that has been generated with a tool to process or analyze it with another tool. Hence, neuroanatomists dealing with 3D morphological reconstructions face the dilemma of having to choose between the functionality and analyses provided by one tool or another. Therefore, current workflows sometimes force users to repeat the work in the different software tools if they want to benefit from the different analyses and capabilities provided by each of them. Proprietary formats and lack of interoperability also make data sharing difficult since the data files are linked to a specific tool.

To the best of our knowledge, none of the existing software tools (open or commercial) offer the possibility of bridging the gap between existing proprietary tools. Thus, the present study focuses on improving interoperability between the two commercial software suites Imaris and NL. These tools have different approaches: NL was conceived as a tool oriented toward neuron tracings, whereas Imaris was designed for 3D use and represents neurons in a three-dimensional way using isosurfaces. Imaris facilitates the reconstruction of dendrites and spines from a particular intensity threshold. It creates isosurface meshes that allow a quite detailed reconstruction of spines. However, to capture the precise morphology of a given spine, it is often necessary to use several intensity threshold isosurfaces. As a result, the file containing a single spine may be composed of several meshes (sub-meshes) that may intersect, leading to inaccurate metrics. To partly overcome this problem, Imaris provides the option to use an extension called Imaris Filament Tracer, which performs a semi-automatic segmentation process that is less accurate than the isosurface tool but that ensures that the spines are attached to their dendrite. Regarding NL, the manufacturer MBF has developed 3D-reconstruction software (NL360), which also makes it possible to semi-automatically reconstruct spines based on intensity thresholds. Although the visualization tools are not optimal, NL360 displays a wide variety of morphometrics to analyze the dendritic arbor.

This article proposes bridging the gap between these two existing proprietary tools to optimize neuron reconstruction workflows. In particular, the tool presented here can create a tracing file that is usable in NL from a neuron extracted using Imaris Filament Tracer. Also, it includes a method to convert the separate sub-meshes into a single corrected unified mesh, based on the method proposed in Eyal et al. (2016). A series of metrics can then be calculated from these unified meshes. The unified meshes can subsequently be visually compared and exported to widely used three-dimensional representation formats and both the meshes and their metrics can be stored in a database and shared.

These improvements have been integrated into the existing Neuronize tool (Brito et al., 2013), giving rise to the second version—Neuronize v2. Improvements have been made with regard to the method of constructing somata, dendrites and spines; new metrics; and comparison of meshes; and a more complete and accurate visualization has also been incorporated.

The following section describes the material and methods, outlining these processes in detail. Three use cases are then described to exemplify the usage and potential of the tool. Finally, we discuss the implications of the contributions and possible impact of the tool.

## Methods and Results

### New Soma Generation Method

Since the first version of Neuronize (Brito et al., 2013), tracing formats have substantially advanced, including new information to describe the neuron more precisely. While in previous versions of the NL ASC format, the soma was represented through a single 2D contour (or a center and a radius), in the new version the soma can be defined by a set of 2D contours describing its 3D shape. The original Neuronize tool could generate a three-dimensional mesh of the neurites from a neuronal path, approaching the soma (which could be incompletely described) from a spherical soma and deforming it based on the beginning of the neuron first-order dendrites, as if they were pulling from the soma.

The application Neuronize v2 includes a new method of generating the soma, based on the new information describing the 3D shape of the soma. The method applies a Convex Hull algorithm to the input set of 2D contours, thus generating an approximated mesh. However, the obtained mesh has irregular triangles and their density is very low. This is inconvenient because the soma mesh will be used later in the deformation process to join the soma with the neurites; hence, the mesh is processed until an isotropic mesh is achieved with a greater density of more uniform triangles. It should be noted that this method has the limitation of only creating convex somata.

Before applying the deformation process to this isotropic mesh, the application checks if there is any neurite that originates from the soma. In these cases, the method assumes that the information representing the soma is correct and removes the tracing points of these starting points, considering the intersection between each neurite and the soma as the real initial tracing point and applying the algorithm accordingly.

However, it is not always possible to retrieve 3D information from NL (due to a low signal to noise ratio). Neuronize v2 can help by including Imaris-generated soma isosurface in the reconstruction to be later imported to NL. Hence, the user can select whether to use: (i) the soma generated from the multiple NL contours; (ii) a soma based on incomplete information (e.g., soma center and radius); (iii) a soma built using exclusively the information about the starting point of the first-order dendrites, following the procedure explained in the first version of Neuronize (when no soma information is available); or (iv) an Imaris surface soma.

### Skeleton Generator

This section describes the method for generating a tracing from the Imaris Filament Tracer file. First, a summary of the components of a tracing is shown, followed by a description of the specific Imaris Filament Tracer format (VRML file) to be exported. Finally, straightforward user instructions about how to export the files are provided.

#### Description of a Tracing

A comprehensive list of the different formats used for neuronal tracings can be found in NLMorphologyConverter: Format Status (NLMorphologyConverter: Format Status). While the tracings accepted by the first version of NL did not include spine information, the newer format of NL ASC can contain spine information as well as a more detailed description of the soma, since it can be defined through a set of 2D contours to describe its shape.

All of these formats include a hierarchy of tracing points. Each tracing point belongs to a particular part of the neuron (soma, axon, apical dendrite, basal dendrite, spine) and the hierarchy between the points can be inferred. Hence, for a tracing point that is part of a neurite, its 3D coordinates, an associated radius (representing the neurite thickness), and its precedent tracing point are defined.

However, the file exported by Imaris Filament Tracer does not have tracing points or a hierarchy between the 3D meshes it describes. In this article, we present a method for generating a hierarchical tracing, in order to be able to obtain NL metrics, including branched structure analysis (comprising segment order analysis), Sholl analysis or vertex analysis, among others.

#### Imaris Filament Tracer VRML Format

Before describing the skeleton generation method, the main particularities of the exported file are outlined to explain the challenges our method faces when unraveling the different pieces of information included in the VRML format.

The VRML file exported from Imaris Filament Tracer needs to be processed before being visualized since it is an old format that some 3D viewers cannot directly process. Note that the files do not contain either the tracing points or the hierarchy between them. Hence, this information needs to be created from the 3D mesh data included in the file.

The VRML format allows the storage of a differentiated set of three-dimensional structures. However, there are some peculiarities in the output files generated using Imaris Filament Tracer exportation. One such peculiarity is that there are identifiers associated with each VRML volume. Identifiers starting with “FilamentSegment6” and “FilamentSegment7” correspond to fragments of dendritic shafts and spines, respectively. Following these prefixes, there is a series of numbers providing an identifier for each volume. The first problem that our method faces regarding the extraction of skeletons is that the volume identifiers are not unique (they are only unique if they belong to the same neurite), so different volumes with the same identifier can be found within the file.

Fragments do not include bifurcations—on the contrary, a bifurcation will be composed of different fragments: the parent fragment which corresponds to the neurite that is going to bifurcate, and a different fragment for each of the “child” neurites, i.e., the neurites resulting from the bifurcation.

**Figure 1:**
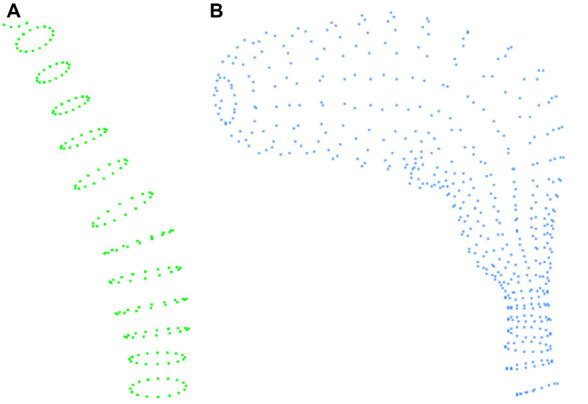
Example of the information contained in the VRML file. **(A)** Example of the slices defining a fragment of a dendritic shaft. **(B)** Slices defining a spine.

In the VRML files, volumes are represented in a rather peculiar way. Neurite and spine meshes are represented by elliptical sections, each defined by a series of points. Thus, each neurite fragment or spine is composed of a series of slices or elliptical sections, with each of these slices in turn formed by 17 points. This allows complete slices of objects to be obtained by simply processing the points of the file in groups of 17 (see Figure 1).

To process these structures, our method starts by reading groups with the three coordinates of a point, and continues until 17 points have been read, providing a slice. In this way, for a particular fragment, we: (i) save the points that form it; (ii) store the number of slices; and (iii) give it a unique identifier. By applying this process consecutively, we read the different fragments in a file. The method then processes the fragments so that, for each slice, it extracts its center, to obtain a tracing point that will be stored with a thickness equal to the slice radius (the distance from the central point to the farthest point of that elliptical slice). Fragments can correspond to dendrite fragments or spines. Hence, each fragment (spine or neurite fragment) is converted into a polyline formed by a set of connected tracing points.

In the VRML file, some spines are defined with two geometrical structures: the main geometry of the spine and a mesh representing a small sphere with a neck that corresponds to the insertion of the spine. This second geometry is probably added by the plugin to prevent spines from appearing disconnected from the neurites or to avoid spines appearing without necks. In this work, only the main geometry of the spine will be considered, since also considering the sphere artificially added by Imaris could lead to errors in future area or volume calculations.

#### Tracing Hierarchy Construction

As mentioned, tracings are typically stored in structured files in which the different tracing points have a hierarchical structure, a preceding tracing point, and a posterior tracing point (unless they represent the termination of a neurite). However, in the VRML file exported from Imaris, the fragments are unconnected since there is no hierarchy.

A method to create a hierarchy starting with the unconnected fragments has been designed. The hierarchy is created based on the proximity of the different polylines representing each fragment. As the VRML file can contain no soma information, the presented method will assume that, for each neurite, the first fragment found is the closest to the soma.

Each neurite is processed independently. Note that, whereas the neurite is highly fragmented in small parts in the Imaris Filament Tracer files, the NL ASC format forces all the tracing points in between two bifurcations (or in between the soma and one bifurcation) to belong to a single polyline. Initially, all polylines are included in a polylines-to-be-processed list. By convention, the first polyline to be extracted from the list for processing will be the polyline representing the fragment closest to the soma, and it will be established as the initial polyline for that neurite.

We defined a connection search algorithm that measures the distance from each point of the polyline being processed to all the points of the rest of the unprocessed polylines. If any of these distance values are below a threshold (called the “connection threshold”), the method assumes that they are connected. Once all of the connections to the current polyline have been found, they are classified and processed depending on their type of connection (we consider three different types of connections), and the current polyline is added to the hierarchy. Next, the method chooses—from amongst the rest of the polylines involved in those connections—which one will be the next polyline to be repeatedly processed. The method continues in this way, processing one polyline at a time, extracting each one from the polylines-to-be-processed list, calculating its connections, and adding it to the hierarchy.

**Figure 2:**
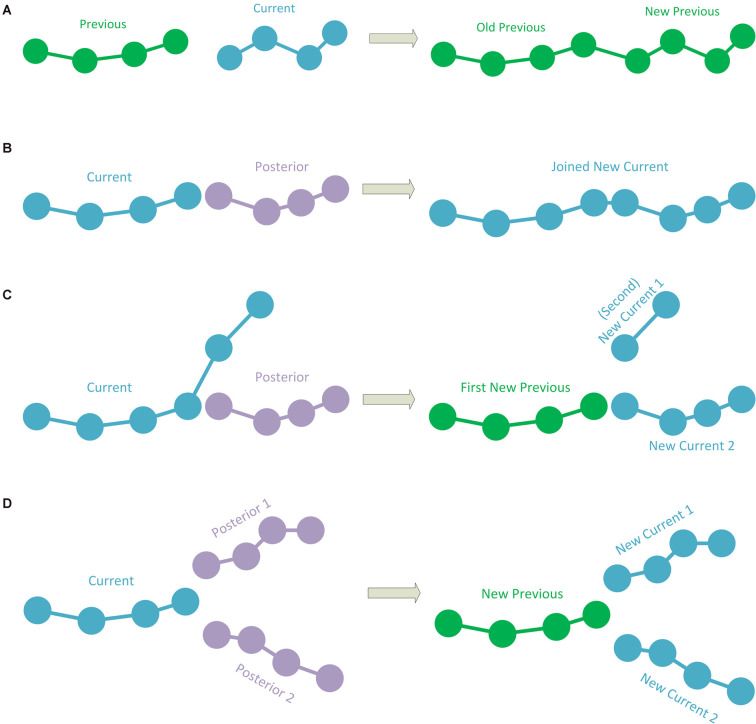
**(A)** A case in which the current polyline does not have posterior connections and is, thus, a terminal fragment. In this case, the current fragment is added to the hierarchy. The green color shows polylines already added to the hierarchy which needs no further processing. **(B)** The current polyline has a posterior connection where the last point of the current polyline corresponds to the first point of the posterior connection. The two polylines are joined into a single polyline and this new polyline is processed (the blue color represents polylines to be processed). **(C)** The current polyline has a posterior connection at an intermediate point. In this case, the current polyline is split into two sub-polylines. The first sub-polyline is added to the hierarchy (green color). The second sub-polyline and the posterior connection are processed (blue color). **(D)** The current polyline has two posterior connections to its last point. The current polyline is added to the hierarchy (in green) and the two posterior connections will be processed (in blue).

**Figure 3:**
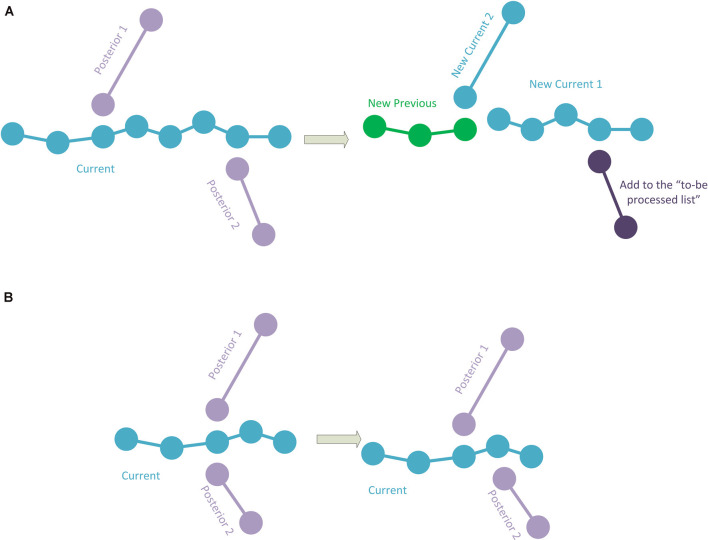
**(A)** The current polyline has two posterior connections at some intermediate points. The current polyline is split into two sub-polylines, and the first sub-polyline is added to the hierarchy (green). The second sub-polyline and the first posterior connection will be processed. Note that the second posterior connection is added to the polylines-to-be-processed list. **(B)** The current polyline has two posterior connections at the same intermediate point. Here, one of the posterior connections is modified to change its initial point to a nearby different point in the current polyline.

Next, the different kinds of connections and how they are dealt with will be explained:

1. The simplest case occurs when the current polyline has no subsequent connection. This implies that it is a polyline representing a terminal fragment. If this is the case, the polyline is simply added to the hierarchy information, according to the previous polyline to which it was connected. To add the polyline to the hierarchy, its starting point is stored as a child of the final point in the last polyline added to the hierarchy (or as the initial point of the tracing if it is the first polyline). Consecutively, the following point after the starting point of the current polyline is added as a child of the first point, and so on (thus, each point of the current polyline has its preceding point as a parent and its subsequent point as a child, except for the last point which will have no child; Figure 2A).
2. When the current polyline has one posterior connection, there are two subcases:
  2.1. If the first point of the posterior connection corresponds to the last point of the current polyline, the posterior polyline is extracted from the polylines-to-be-processed list and the two polylines are then joined into a single one. This newly joined polyline will be the next current polyline to be processed (Figure 2B).
  2.2. If the first point of the posterior connection corresponds to an intermediate point of the current polyline, the current polyline will be divided into two sub-polylines. The first sub-polyline, from the starting point to the point of connection is added to the hierarchy. The method will continue repeatedly processing both the second sub-polyline (starting at the connection point and finishing at the final point of the original current polyline) and the posterior connection polyline (Figure 2C).
3. If the current segment has two posterior connections, whose initial points correspond to the final point of the current polyline, which means that the current polyline will bifurcate into two: the current polyline will be added to the hierarchy information, according to the previous polyline that was connected to it. The method will continue repeatedly processing the two posterior polylines (see Figure 2D).
4. If there are two posterior connections, where—for at least one of the posterior connections—its initial point corresponds to an intermediate point of the current polyline, a specific procedure is applied. The points in the current polyline corresponding to the starting points of the posterior connections are calculated. Among them, the method chooses the connection point closest to the initial point of the current polyline. This connection point will be used to divide the current polyline into two parts as in the case of the polyline of Figure 2C, where the first part will be added to the hierarchy. The method will then repeatedly continue with the second part of the divided current polyline and with the first posterior connection (that will be removed from the polylines-to-be-processed list), whereas the polyline of the second posterior connection will continue to be in the polylines-to-be-processed list (Figure 3A). In the event that the two (or more) posterior connections correspond to the same intermediate point of the current polyline (or that there are three or more connections if it is a fragment final point), a pre-process will be applied to match the initial point of one of the posterior connections to another existing nearby tracing point in the current polyline. As a result, each posterior connection has its own corresponding point, and then the method proceeds as explained (Figure 3B).
5. When there are more than two posterior connections, the problem is simplified by treating each of the posterior connections, in turn, starting with the connection closest to the initial point of the current polyline, by successively subdividing the current polyline as in the previous cases. Again, if two (or more) posterior connections have the same corresponding point, a preprocess is applied (as in Figure 3B).

This method assumes that fragments are constructed from the initial part of the dendrite towards the endpoints, i.e., from the soma towards the end of the dendrites. However, this is not always the case in the exported files and some fragments are inverted, with their initial point further from the soma than their final point. These inverted fragments caused problems in the algorithm, and NL ASC does not support this method of construction. To solve this, a stage has been added just after the connection search in which, if the connecting point is located on the second half of a polyline representing a fragment, it is detected as being inverted and it is realigned before continuing normally with the algorithm.

This method is repeated for each neurite in the neuron until the complete hierarchy has been built. Since the geometry provided by the VRML file has some intersection zones between the consecutive fragments, the hierarchy computed can sometimes present repeated points. To solve this problem, the resulting hierarchy is post-processed by traversing the neurites and eliminating repeated points (points closer than a threshold).

#### Connection Threshold

This configuration parameter determines the maximum distance threshold (in microns) between two dendrite fragments for them to be considered connected. Note that a connection threshold value that is too small can cause some dendrite fragments to be disconnected because they are far from the others. On the contrary, a value that is too large will ensure that no dendrite fragment is unconnected, but this could cause problems such as erroneous connections and bad management of bifurcations; therefore, a value that is too large is not recommended.

For usability reasons, the tool automatically searches an appropriate value for the connection threshold parameter. It starts with a small value and progressively increases the value if unconnected fragments are still found, re-processing the tracing generation.

#### Soma Generation for the Tracing

Once the dendrite hierarchy and geometry have been calculated and are fully described, it is necessary to calculate the points that define the soma. However, as mentioned above, the soma may come from Imaris or, if the input file does not contain any information related to soma, it is necessary to make an approximation from the available information. In the latter case, the soma will be approximated starting with a sphere, where its center is calculated as the barycenter of the initial points of the first order neurites. The radius will be the shortest distance between the newly calculated center and the beginning of the first order neurites.

Finally, it is necessary to transform the point-radius sphere representation used, or the 3D mesh obtained from Imaris, to convert it into a representation based on a set of 2D contours (since this representation is supported by the NL ASC format). Note that this soma will later be deformed by the tool as in Brito et al. (2013) and the set of 2D contours will be updated.

#### Adding Spines to the Tracing

Finally, once the dendrites and the soma have been generated and described, an algorithm will add spines to the tracing. The spines can be provided in a geometry Filament Tracer file or an Imaris file containing the spine skeletons as polylines.

If the input file is an Imaris Filament Tracer file, the spines’ skeletons are calculated analogously to the above-mentioned neurites’ skeletons calculation. However, if the Imaris Filament Tracer file does not contain spine information, but the user has provided—as an input file—an Imaris file that contains measurements of the spine lengths (manually measured from the point of insertion in the dendritic shaft to the distal tip of the spine), spines can also be added. In order to process this file, for each spine length, the first point will be considered the spine’s head, and the final point will be considered the spine’s insertion point.

Once the spine information has been obtained, spines are hierarchically added to the tracing. For each spine, the distances from the spine’s insertion point (the beginning of the spine) and all the points from the tracing are calculated. If the minimum distance calculated is below the connection threshold, the spine will be connected to the tracing point from which this minimal distance was calculated.

One limitation of the previous version of the tool was that the tracings file format that was compatible with it could not contain spine information, so the tool placed spines (procedurally built or from a specified folder) in a random orientation—determining their position following a given distribution function. As previously mentioned, the current NL ASC format contains spine information. Thus, spines are represented by a single point, corresponding to the end of the spine, and the insertion point of each spine is considered to be the previous point within the tracing (where a spine is found). Note that this may be slightly less accurate than adding the insertion point of the spine to the tracing but doing this would involve including new tracing points, which is rather unnecessary given that the deviations are minimal.

#### How to Export Files to be Used for Tracing Generation

Finally, we briefly explain how users should export the files from Imaris Filament Tracer. If there is an apical neurite, users should export the whole apical neurite with all of its branches (and spines, if any) in the same file. The rest of the neurites including the axon can be exported to a single file or, if desired, to different files, as long as each file contains a complete dendritic branch or axon that departs from the soma. For our method to work properly, the first point of each neurite should be as close to the soma as possible. If the user wants to automatically generate several neuronal tracings at once, more detailed instructions can be found in the user manual attached as Supplementary Material.

Regarding spines, the new information available in the NL ASC tracing files refers to their position and orientation. Hence, spines are exported as a small line that connects the tracing to a point representing the end of the spine.

### New Placement of Spines

In the first version of Neuronize (Brito et al., 2013), there was no information regarding the geometry of the spines, so spine geometry was obtained from a series of spines that were included in Neuronize by default. The spine placement process consisted of placing it in the corresponding tracing point (calculated following a distribution function) with a random orientation.

In the version presented here, in addition to a more detailed soma, the NL ASC files contain spine information, where each spine is represented by a small line that defines the spine placement and its orientation. Note that some neuroscientists could mark the end of the spine inaccurately causing the small line representing the spine to also be inaccurate. A spine geometry from a small local database will be randomly selected to be placed according to the small line defining the spine within the NL ASC tracing. These spines are centered on the origin and aligned with the Y-axis and are transformed to match the small lines.

Furthermore, if the input file is an Imaris Filament Tracer file rather than a NL ASC file, the actual geometry of the spine is already in its position and orientation. Hence, we process the file to: (i) extract the tracing; (ii) create its corresponding 3D mesh; (iii) store the spines in a database (transforming them to be centered on the origin and aligned along the Y-axis); and (iv) place the geometry of these spines in their real position and orientation.

In addition, the user can provide the real geometry of spines generated with Imaris isosurfaces, and add either the original or the repaired spines to the visualization. Note that, in this case, the spines are also added to the database without applying any transformation, so they cannot be added to other neurons.

### Geometry Unification and Repair

As mentioned, the meshes provided by Imaris may contain certain errors, such as faces that intersect and/or holes, which can cause errors in the metrics obtained from these meshes—for example, errors in the area and the volume of a mesh.

To deal with this problem, a geometry corrector is provided to repair geometries and to recalculate the metrics from these repaired geometries. The geometry corrector also allows the meshes (both the original ones and the repaired ones) to be exported to widely used 3D formats, thereby facilitating the sharing of data with other neuroscientist laboratories.

To repair the meshes, the first step involves reading them. The tool accepts both VRML and IMX formats. The VRML files provided by Imaris contain a lot of useless information and inaccurately-formed structures that make it impossible to open the file with some standard programs. Therefore, an algorithm has been developed that builds a new “clean” VRML file which contains only the relevant sections from the original VRML file, that is, the geometry of spines and neurite branches.

In addition, the IMX files contain a lot of information that is not related to the geometry of the neuron. However, the format does not include unique tags indicating which element a given geometry is representing. For example, there is no tag to define a spine, so the importing algorithm will assume that the geometry represents a spine by inferring it from the number of vertices. When a geometry that has between 10 and 10,000 vertices is found in the file, the algorithm assumes that it is a spine. Once all relevant geometries of the file have been obtained and tagged, they are included in a clean VRML file (so the following steps are common regardless of whether the clean VRML comes from a processed VRML or a processed and transformed IMX). Next, each mesh contained in the processed VRML file is repaired separately. The repair process is based on the one proposed in Eyal et al. (2016), but it has been extended to repair not only spine meshes, but also neurite fragment meshes and soma meshes. First, the mesh is voxelized, thus moving from a surface representation to a volumetric representation. A dilation-erosion process is then carried out on this volumetric representation to join disconnected geometry (such as a spine and its neck if they were separated). Next, a Gaussian smoothing is performed and, finally, the three-dimensional representation is converted back to a surface representation using the Marching Cubes technique (Lorensen and Cline, 1987). Since the repair of neurite fragments presented memory problems, a method that calculates the Object Oriented Bounding Boxes of the mesh using Principal Component Analysis (Dimitrov et al., 2006) has been implemented, thus significantly reducing the number of voxels to be dealt with by the algorithm. Additionally, some options are available to customize the repair process that, by default, is configured for spines.

Upon completion of the repair process, new metrics are calculated on that mesh (such as area, volume, and mass center) and stored in a .csv file that contains these metrics for each mesh contained in the input file. The user can then store both the original meshes and the repaired meshes in separate files with standard formats.

### A Tool to Compare Meshes With Hausdorff Distance

**Figure 4:**
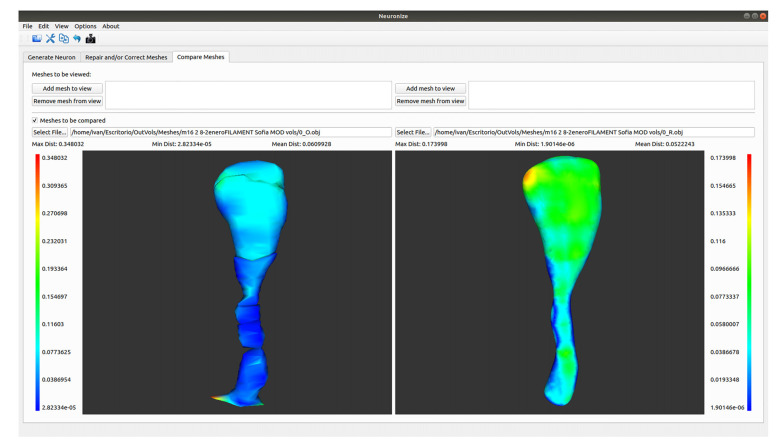
Differences between the spine meshes of the same spine before (left) and after being repaired (right).

A comparison functionality has been added to the tool (see Figure 4). As a result, structures obtained with different methods (like a soma based on a sphere or based on a set of 2D contours) or unrepaired and repaired meshes can be compared.

The first option allows different meshes to be visualized side by side. Using this option, both meshes will be single-colored. However, the comparison tool also offers the interesting option of showing the differences between two object meshes in different colors. More specifically, it displays two visualizations side by side in which the meshes are colored using a transfer function based on the Hausdorff distance (Cignoni et al., 1998) between the meshes. This transfer function is visible on the interface, together with the associated distance values, to allow the user to estimate the magnitude of the difference.

To facilitate navigation, both visualizations are coordinated, so that any camera movement in one of them is also reflected in the other. The tool also provides a series of metrics based on this distance, such as the average error, maximum error, and minimum error.

### Improving Data Sharing

The use of proprietary tools hinders data sharing between teams since the data is linked to specific proprietary software. To overcome this limitation, the new version of the tool includes a small local database that stores all the processed and extracted information of the neuron in widely used standard formats. In this way, the generated soma, the extracted spines and the reconstructed meshes can be stored, together with some description metrics (such as area, volume, and mass center). This database will facilitate the storing and sharing of models, and the tool also includes the option to reuse spine models from the database in the event that no spines are available.

To store the models in the database in a generic way, the geometry of the spines are stored centered on the origin and aligned with the Y-axis, while the position and orientation of each spine in a particular neuron are independently stored.

Since the spines present in the archives of Imaris Filament Tracer are located in their real positions, after processing the file, it is necessary to transform them. First, a series of rotations are applied to a spine to align it with the Y-axis. These rotations are calculated taking into account that all the spines from the input file have a flat base. Thus, the normal vector to this base determines the actual orientation of the spine, which is transformed to coincide with the Y-axis. Once the spine is correctly oriented, it is moved to the origin, so that the midpoint of the base is at the origin. These transformations are stored within the database as a rotation (through a quaternion) and a translation (through a vector).

To reuse spines from the database, the application takes the geometry and applies the corresponding transformations (based, for example, on the small lines describing spines contained in a NL ASC tracing file, or placing the spines following a distribution function). Thus, to facilitate the sharing of data between different neuroscientist laboratories, a database is included in this new version, to store information from all the neuronal data processed by Neuronize in open standard formats. This eliminates the dependence of the proprietary formats that lead to the data only being compatible with a specific tool. Using this database, Neuronize v2 can help to create a local spine repository that is built up from the processed spines (the repaired spines from traditional Imaris or the spines with necks from Imaris Filament Tracer) for future reuse.

## Use Cases

This section includes three examples of how to use the tool.

### Use Case 1

**Figure 5:**
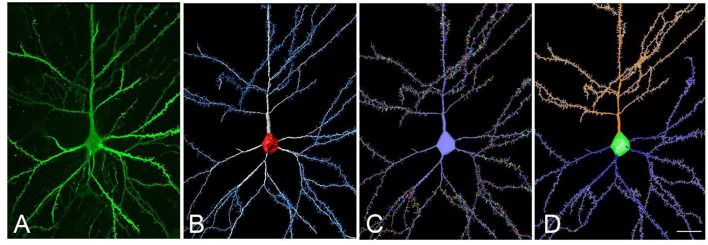
**(A)** Confocal microscopy image of an intracellularly injected human layer III pyramidal neuron. **(B)** Three-dimensional reconstruction of the morphology of the cell shown in **(A)**, using Imaris. **(C)** The building of the soma, dendrites, and spines from the neuron shown in **(B)**, using Neuronize. **(D)** Neurolucida visualization of the neuron shown in **(C)**. Scale bar (in **D**): 20 μm in **(A–D)**.

Here we present an example to obtain a tracing file usable in NL from a neuron extracted using Imaris Filament Tracer (Figure 5). The proposed workflow would be as follows: (a) reconstruct dendrites and spines using Imaris Filament Tracer; (b) go to *Neuronize v2/create a neuron* and import VRML files to obtain a tracing (ASC file) from the 3D representation imported from Imaris Filament Tracer, which was semi-automatically created. The steps the tool performs to obtain the ASC file are automatic. In this stage, there is also the option to create a soma *via* the software if desired; and (c) The created ASC file can be directly opened by NL and a variety of analyses can be performed, including, for example, branch order analysis or Sholl analysis (which analyses the branching structure of dendrites and spines). These steps are completely automatic, avoiding long, tedious and error-prone processes.

### Use Case 2

**Figure 6:**
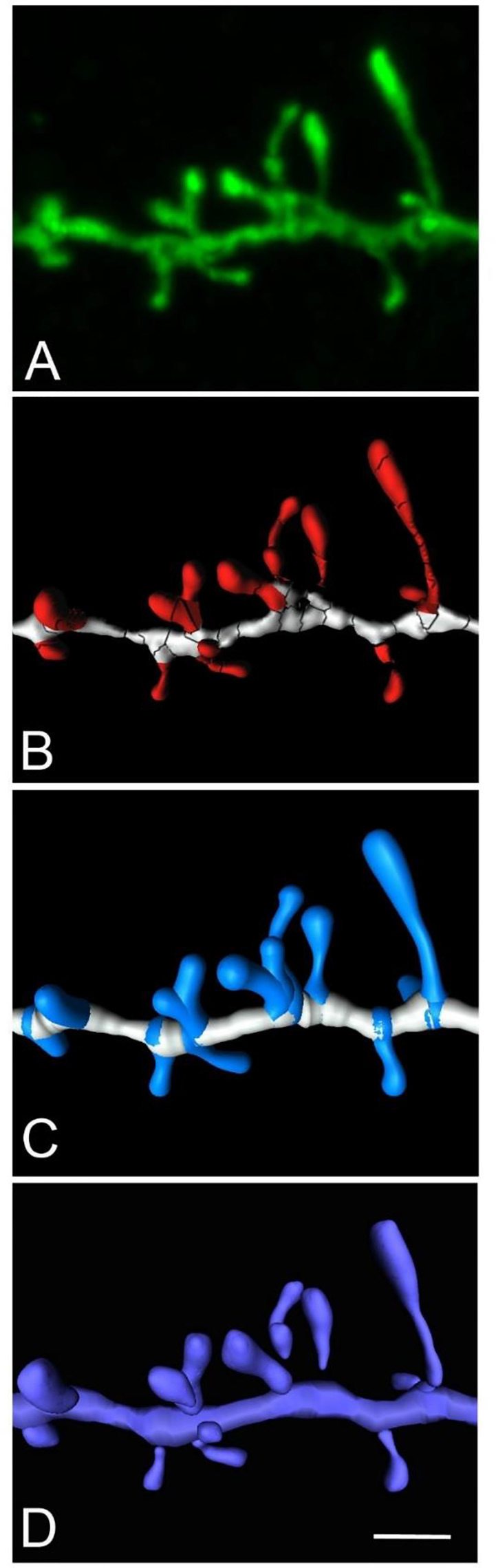
High magnification image of a human basal dendritic segment from an intracellularly injected layer III pyramidal neuron. **(B,C)** Three-dimensional reconstruction of the morphology of each dendritic spine shown in **(A)**, using Imaris isosurfaces **(B)** and Imaris filament tracer **(C)**. **(D)** The building of the spines from the reconstruction is shown in **(B)**, using Neuronize after the unification and repair process. Scale bar (in **D**): 2 μm in **(A–D)**.

Here we present an example to obtain a continuous unified spine mesh extracted from several disconnected Imaris isosurfaces (Figure 6). The proposed workflow would be as follows: (a) reconstruct spines using the Imaris Isosurface tool, which captures great morphological detail, although it is often necessary to use several surfaces of different intensity thresholds to capture the complete morphology of a dendritic spine. As a result, one particular spine may be composed of several disconnected meshes; (b) go to *Neuronize v2/unify and/or correct meshes* to directly obtain the 3D unified meshes (OBJ files). This step provides the surface area calculation of the unified mesh (XLS file).

### Use Case 3

**Figure 7:**
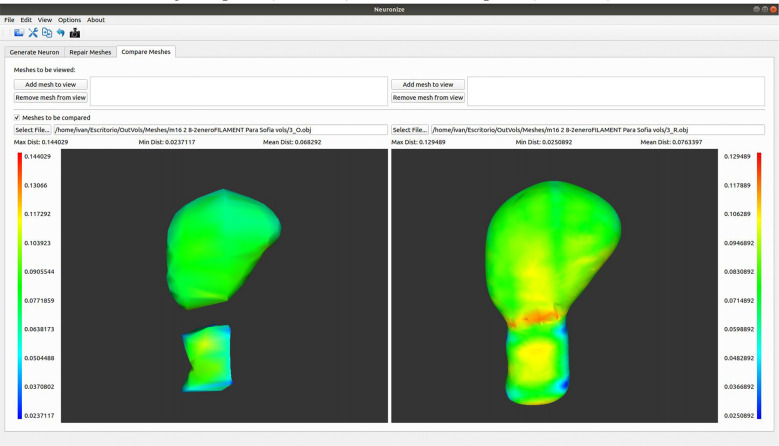
Graphical user interface of Neuronize showing a high magnification image of a human dendritic spine before (left) and after (right) the unification and repair process. The larger variations in the meshes are represented in warm colors (reds, oranges, and yellows).

Here we present an example to visually compare meshes (Figure 7). As a next step following on from the steps outlined in use case 2, it is possible to go to *Neuronize v2/compare meshes* and obtain the 3D visualization of the original spines (OBJ files) and the unified spines (OBJ files).

## Discussion

This article presents Neuronize v2, a tool designed to bridge the gap between different existing proprietary tools, in order to modify and optimize neuroscientific workflows. This has been achieved by processing the output data from proprietary tools, extracting the relevant information, transforming it, and offering functionalities to correct, store and share the data in standard formats, making it available to all users. The tool was developed in C++ and python and has been released for Linux, Mac OSX and Windows operating systems. It is publicly available at https://vg-lab.es/neuronizev2/.

This tool introduces modifications in the current workflows that allow users to optimize the use of commercially available tools by sharing the extracted data of one tool with other tools. Specifically, users will be able to directly generate a complete tracing compatible with Neurolucida from the data obtained with Imaris Filament Tracer, thus avoiding the need to extract data from neurons with both tools to obtain the metrics and analyses that each tool provides. VRML files exported from Imaris Filament Tracer can be read and deciphered to automatically extract the meshes and the relevant information.

Also, the tool processes the meshes to obtain the position and orientation of spines if they are present in the file. If the spines are not present in the file, this information can be taken from Imaris files that contain measurements representing the length of the spines.

Another new feature of this version of the tool is its capacity to process the meshes provided by Imaris. Although Imaris is highly accurate, it is often necessary to use several surfaces of different intensity thresholds to capture the complete morphology of a particular structure (i.e., spine). Thanks to this repair and unifying process, it is possible to calculate more precise metrics of the structure. The tool also allows the meshes—both unified and original—to be stored in standard 3D formats, allowing the sharing of data and new metric calculations.

The usage of software tools for the reconstruction of neurons is widespread today although pioneer systems for 3D dendritic tracing were developed decades ago, before the appearance of NL system (e.g., Overdijk et al., 1978). These early methods have been very useful to improve the analysis of neuron morphology (e.g., Mrzljak et al., 1992; Koenderink et al., 1994; Petanjek et al., 2008). According to the literature focusing on new 3D meshes generated from existing tracings, regarding the soma, some tools are based on the deformation approach proposed in Neuronize (Brito et al., 2013). For instance, NeuroMorphoVis (Abdellah et al., 2018) uses the same approach as the first version of Neuronize to generate a soma from incomplete information, whereas NeuroTessMesh (Garcia-Cantero et al., 2017) uses a similar method, but replaces the mass-spring deformation with a Finite Element Method deformation, which is easier to parametrize and generates a smoother membrane surface. However, none of these tools can make use of the new information about the soma present in Neurolucida files, which contain a set of 2D contours that Neuronize v2 uses to generate a more accurate soma.

Regarding the generation of the mesh, NeuroTessMesh (Garcia-Cantero et al., 2017) allows the visualization of complex scenarios with several neurons, reducing the resolution of the distant neurons, by using a GPU-based approach that allows dynamically-adaptive multiple levels of details (LOD) when visualizing the mesh. NeuroMorphoVis (Abdellah et al., 2018) first applies multiple processes to the input tracing to remove artifacts that can negatively affect the generated mesh. It then uses a new meshing algorithm that allows the generation of a unique neuron mesh from a set of watertight meshes with the advantage being that the mesh vertexes are related to the original tracing points. However, none of the studied tools that generate 3D meshes from tracings have the capacity to add spines to the visualization.

Regarding spines, Neuronize v2 uses the available spine information contained in the input files to add the spines to the representation. If there is spine information in the NL ASC files, this information is used to place the spines in their real position and orientation. However, if the spine information comes from Imaris Filament Tracer files, the application will place the real spine geometries in their position and orientation. If the Imaris Filament Tracer file does not contain spine information, the spine position and orientation could be taken from an Imaris default file if provided. Alternatively, the geometry of the spines, as well as their position and orientation, can also be obtained from an Imaris isosurfaces file and the spines can then be placed in position in their appropriate orientation. Note that the spine meshes created with Imaris isosurfaces could be previously unified and/or repaired with the tool if required.

Besides the improvements made to include the spines, the main contribution of Neuronize v2 is that, to our knowledge, it is the first tool that allows the gap to be closed between Imaris and NL, making it possible to benefit from the data extracted from Imaris. *Neuronize v2* obtains a tracing (ASC file) from the 3D representation imported from Imaris Filament Tracer, which was semi-automatically created. The steps the tool performs to obtain the ASC file are automatic. This tracing can then be opened directly to make use of all of the analyses available in Neurolucida.

As a result, branch order analysis or Sholl analysis can then be extracted. The detailed segmental and topological analysis of different compartments within the dendritic tree is crucial to find subtle morphological differences, which cannot be obtained by analyzing general data about neuron morphology (e.g., Groc et al., 2003; Benavides-Piccione et al., 2020).

Finally, being able to save the models in a generic database using standard 3D formats greatly facilitates data sharing between different research groups within the scientific community, since the limitation of owning commercial tool licenses is eliminated.

In the future, we plan to continue exploring new functionalities that serve as a link between existing tools, to make the most of the benefits of each of the software programs, overcoming software dependencies and limitations—to propose new, more efficient workflows and make the tools open access. We are also working on an integrated framework in which different tools can interact with coordinated views and be connected, exchanging information and data between them.

## Data Availability Statement

The raw data supporting the conclusions of this article will be made available by the authors, without undue reservation.

## Author Contributions

RB-P, IF-E, and JD conceptualized the idea of bridging the gap between existing proprietary tools, acquired the data, tested the tool, and validated the correctness of the obtained results. IV designed and implemented the new features of this new version. PT implemented the first functional proof of concept of the Skeleton Generator algorithm. JB developed the first version of the tool and proposed to use the new information about the soma in Neurolucida to achieve more accurate shapes. SM, LP, and SB proposed and designed some of the new features. RB-P, IF-E, SB, and IV made the illustration of the manuscript. SB directed the entire process of implementation and design of the new functionalities in this publication. SB and IV made a first draft of the article. All authors contributed to the article and approved the submitted version.

## Conflict of Interest

The authors declare that the research was conducted in the absence of any commercial or financial relationships that could be construed as a potential conflict of interest.
