# Supplementary material for: Neuronize v2: Bridging the Gap Between Existing Proprietary Tools to Optimize Neuroscientific Workflows
Source: Front Neuroanat. 2020 Oct 6;14:585793. doi: 10.3389/fnana.2020.585793 (PMC7646287; doi:10.3389/fnana.2020.585793)
Supplement: Supplementary file 1 [file Data_Sheet_1.PDF]

Neuronize V2 User Guide

1 First-time execution ... 1
2 Neuron Generation ... 1
2.1 Generation of a single neuron ... 2
2.2 Generation a set of neurons. ... 9
3 Mesh Repair ... 10
3.1 Installation and use requeriments ... 12
4 Comparison between two meshes ... 12
5 Export information from the database ... 13

Neuronize v2 is an application that helps in the process of data acquisition workflows by providing interoperability functions to generate neuronal tracings. It allows using the 3D data extracted with Imaris Filament Tracer to automatically generate a tracing with dendritic spine information that can be read directly by Neurolucida. It also allows to build 3D meshes from the tracing files. It is also possible to unify and/or correct ill-formed meshes (i.e. dendritic spines) and to compare meshes.

1 First-time execution

The user needs to unzip the folder and then double click on "neuronize.exe". If the application does not open, the user should execute the file ("vcredist_x64.exe"). Once this is done, the user will double click on "neuronize.exe" again. Note that, if the user wants to use the mesh repair function, Python 3 must be installed from:

https://www.python.org/ftp/python/3.7.4/python-3.7.4-amd64.exe

2 Neuron Generation

To generate one or more neurons from tracing files or from Imaris Filament Tracer files, the user needs to open the "Generate Neuron" tab (see Figure 1). Once in this tab, the user can select between generating a single neuron "Generate One Neuron" or generating multiple neurons at the same time, clicking on the "Generate a set of Neurons" button.

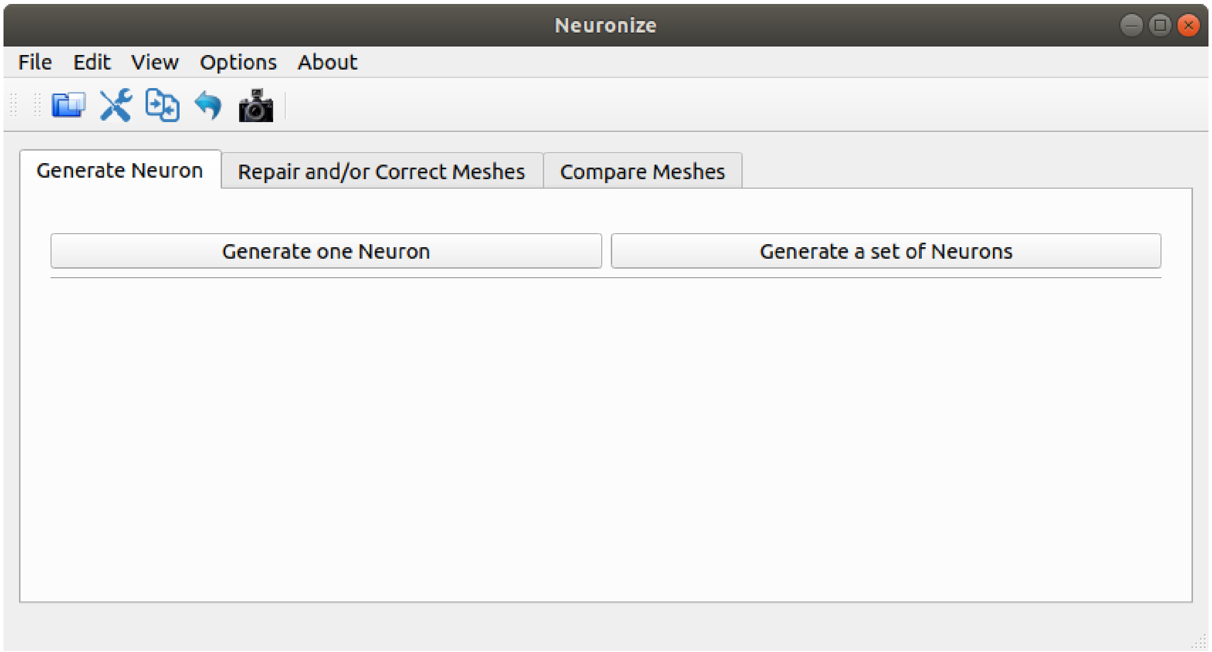

Figure 1. Interface to generate a tracing file from Imaris Filament Tracer VRML files. The user can generate one single neuron following several steps or automatically generating a set of neurons from a folder**.**

2.1 Generation of a single neuron

First, it will be explained how to generate a single neuron. The user can choose as input file three different types: tracing files in SWC format, tracing files in ASC format or exported Imaris Filament Tracer files (VRML format) (see Figure 2):

a) If a user wants to load a tracing file (either ASC or SWC), the user selects that option and presses “Next”, then opens the desired tracing file, and finally, selects the output folder where all the 3D neuron will be exported. .
b) If,on the contrary Imaris Filament Tracer files are to be used as input, the user selects the VRML option and presses next ;
    i. Now the user should select the desired VRML files to process. Note that if there is an apical dendrite in the input neuron, the complete apical dendrite must be provided in a single file and all the basal dendrites can be provided in a single file or using one file for each basal dendrite. Once all the desired files are indicated, the user presses “Next” (see Figure 3).
    ii. In this step, if there is an apical dendrite, the user should select, from the loaded files, the file that contains it and press “Next”. If the neuron doesn't have apical dendrite, the user just presses “Next” without selecting any file. (see Figure 4).
    iii. Now, the user can provide a file that contains an Imaris soma to be added to the generated Neuron. Note that this step is only available if the user has Python 3.7 installed in the system.
    iv. If the tool detects that the files provided do not have spines, the tool allows to add the spines as Imaris lengths files (that contain one polyline per spine, where the first point

is considered the head of the spine). Please, keep in mind that this step only applies when no spines are found..

v. The last step is to select an output folder, where all the generated information will be exported.

Once the process of selecting the input files is finished, the tool continues with the step of generating the soma mesh. (see Figure 5).

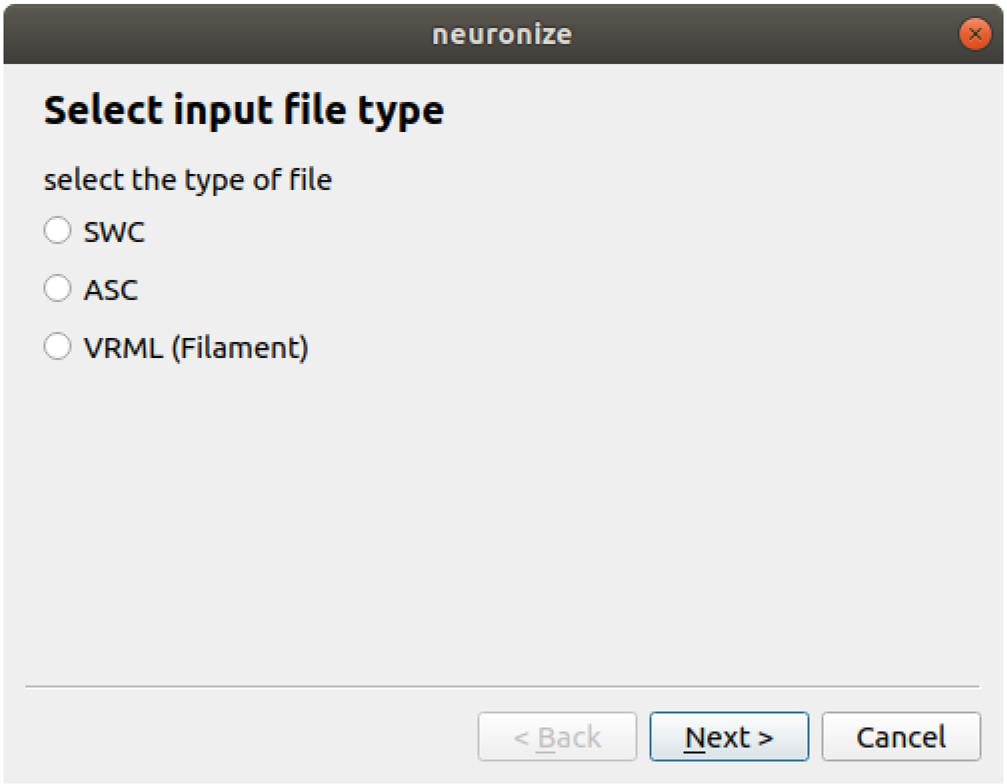

Figure 2. Load dialog that allows selecting between the different supported formats.

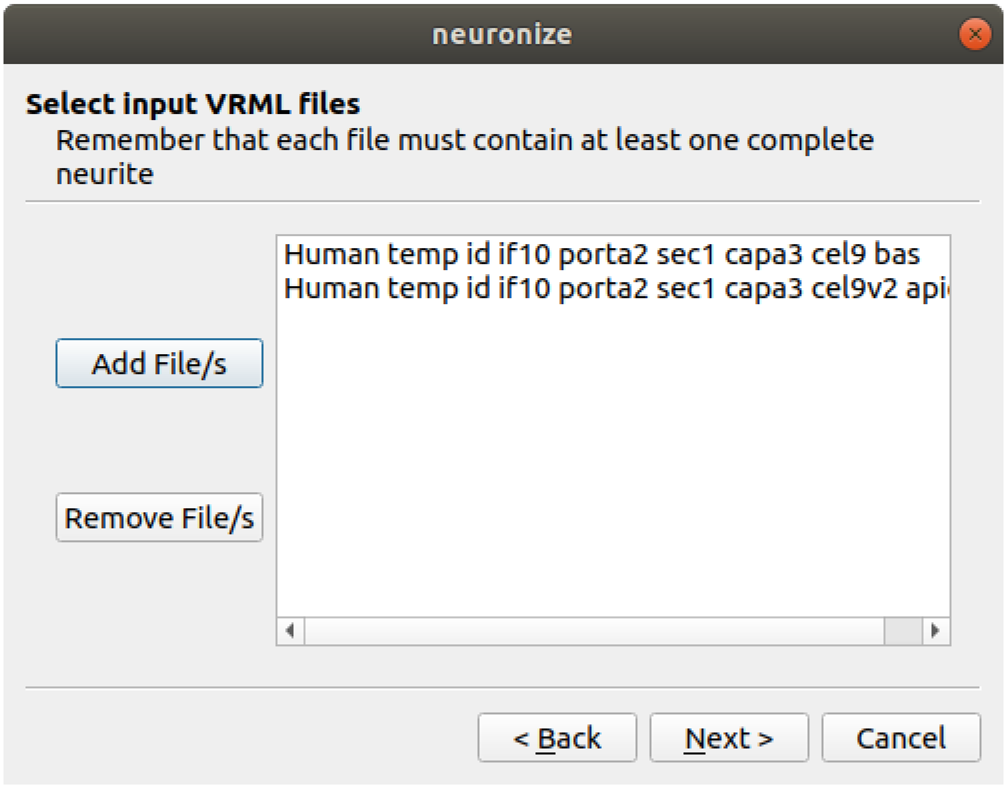

Figure 3. Window to load the desired VRML files corresponding to a neuron.

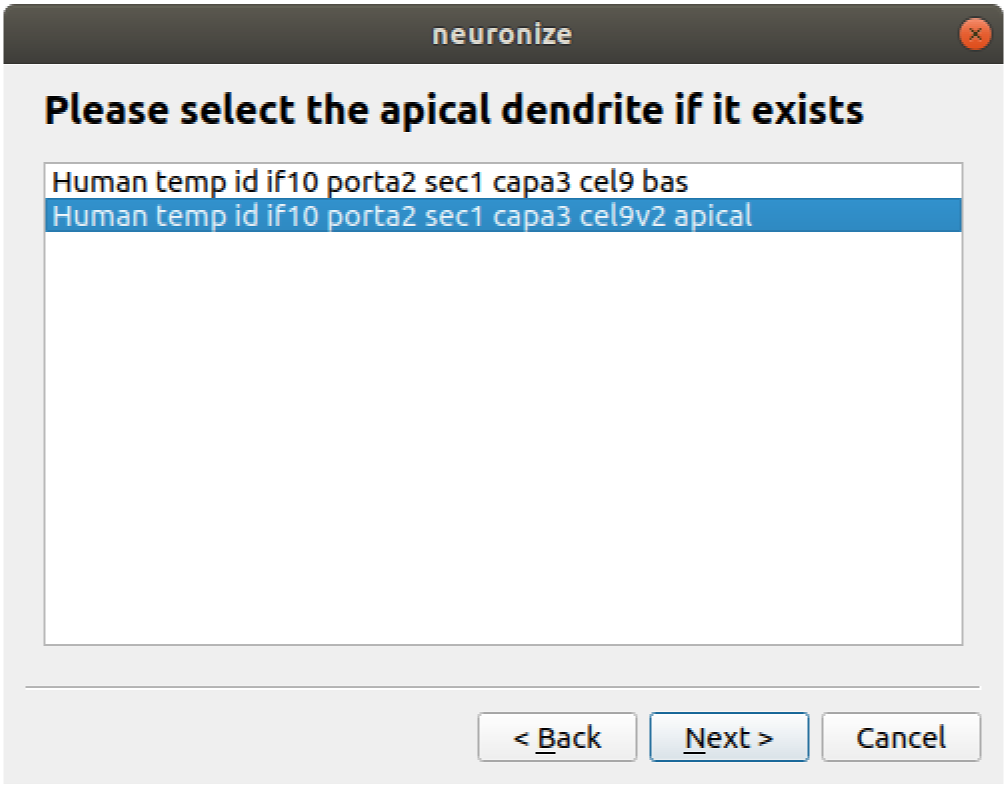

Figure 4. Interface to select the file that contains the complete apical dendrite.

In this step, the neuron soma is automated constructed from an initial shape of the soma, that starts deforming until a dialog indicates the finalization of the deformation process. Then, the user clicks on the “Go to Dendrite Generation” button to continue to the next step (see Figure 5).

Note that, when the selected input file is a Neurolucida file with a set of 2D contours describing the soma information, or if an Imaris soma is provided, those descriptions could occasionally be incorrect, leading to the generation of an unrealistic soma. If this is the case, the user can decide to ignore this information and to use a spherical soma as a first approximation of the shape for the deformation process by clicking on “Use Spherical soma” Button. Note that this button only appears in the mentioned cases.

If the generated soma is not satisfactory, the user can press the button “Rebuild (advanced options)” to get an advanced configuration of the soma generation process (see Figure 6) to achieve a better result. This advanced configuration has two parameters:

- Soma scale factor: This parameter allows to control the size of the initial soma sphere. This parameter is defined as the percentage of the initial size, for example, a value of 1,1 makes a soma 10% bigger than the initial soma.
- Uncollapse springs %: This parameter allows to control the influence of neurites on the whole mesh. Higher values maintain the initial spherical shape and cause more sharp dendrite deformations.

Once the parameters are configured, press the button “Build Soma” to apply them.

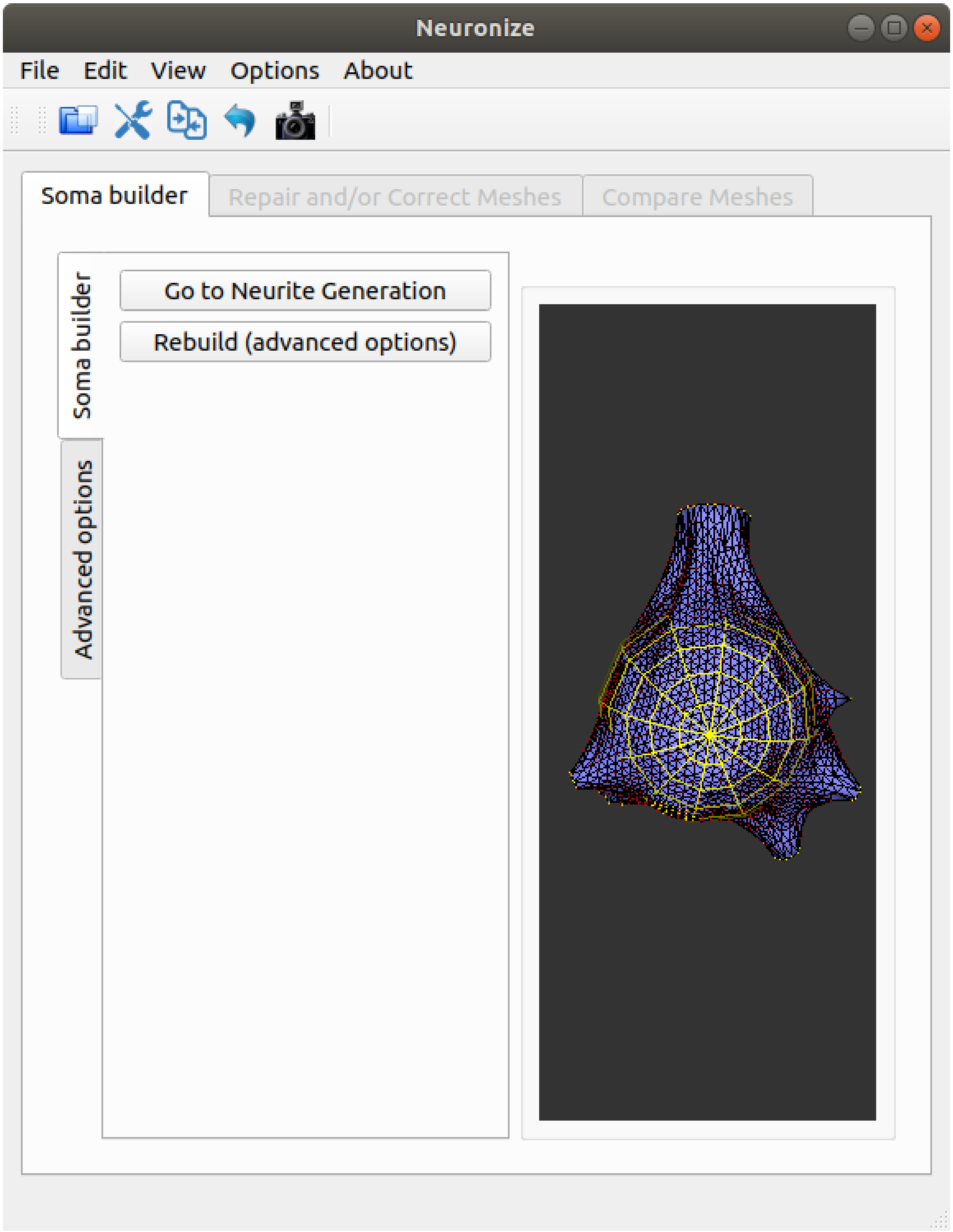

Figure 5. Interface inside the stage of "Soma builder" in which the result is displayed once the soma has been generated. Clicking on the "Go to Neurite Generation" button will proceed to the next step.

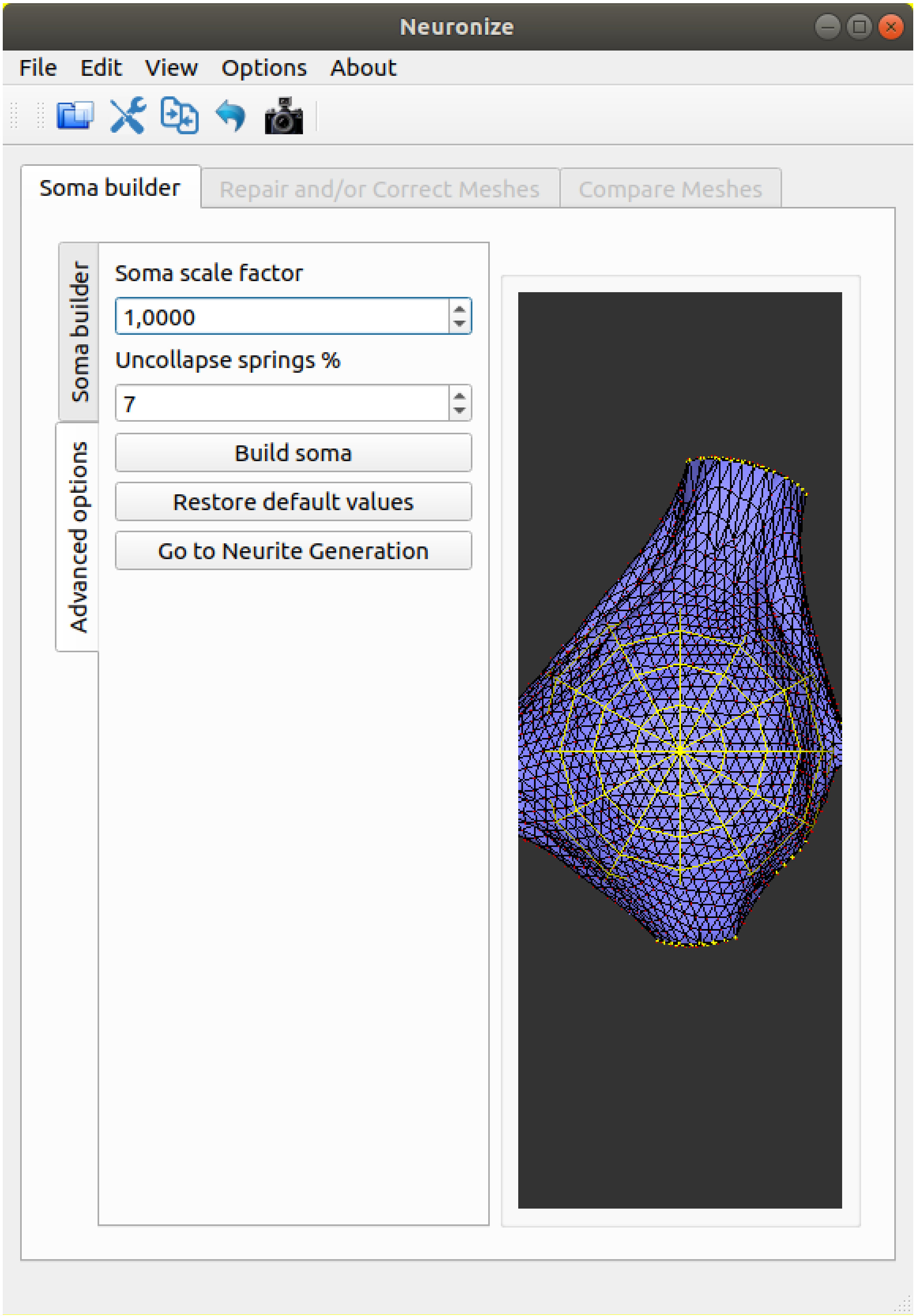

Figure 6. Interface for the advanced configuration of the soma deformation process**.**

The next step of the algorithm automatically starts generating the dentrictics branches. This process, if the input files come from Imaris Filament Tracer, may require a considerable amount of time. Once the tool has finished generating the neurites, it is possible to smooth the geometry obtained through the "Smooth" button. Finally, the "Go to Spine Generation" button is clicked to continue with the generation of spines (see Figure 7).

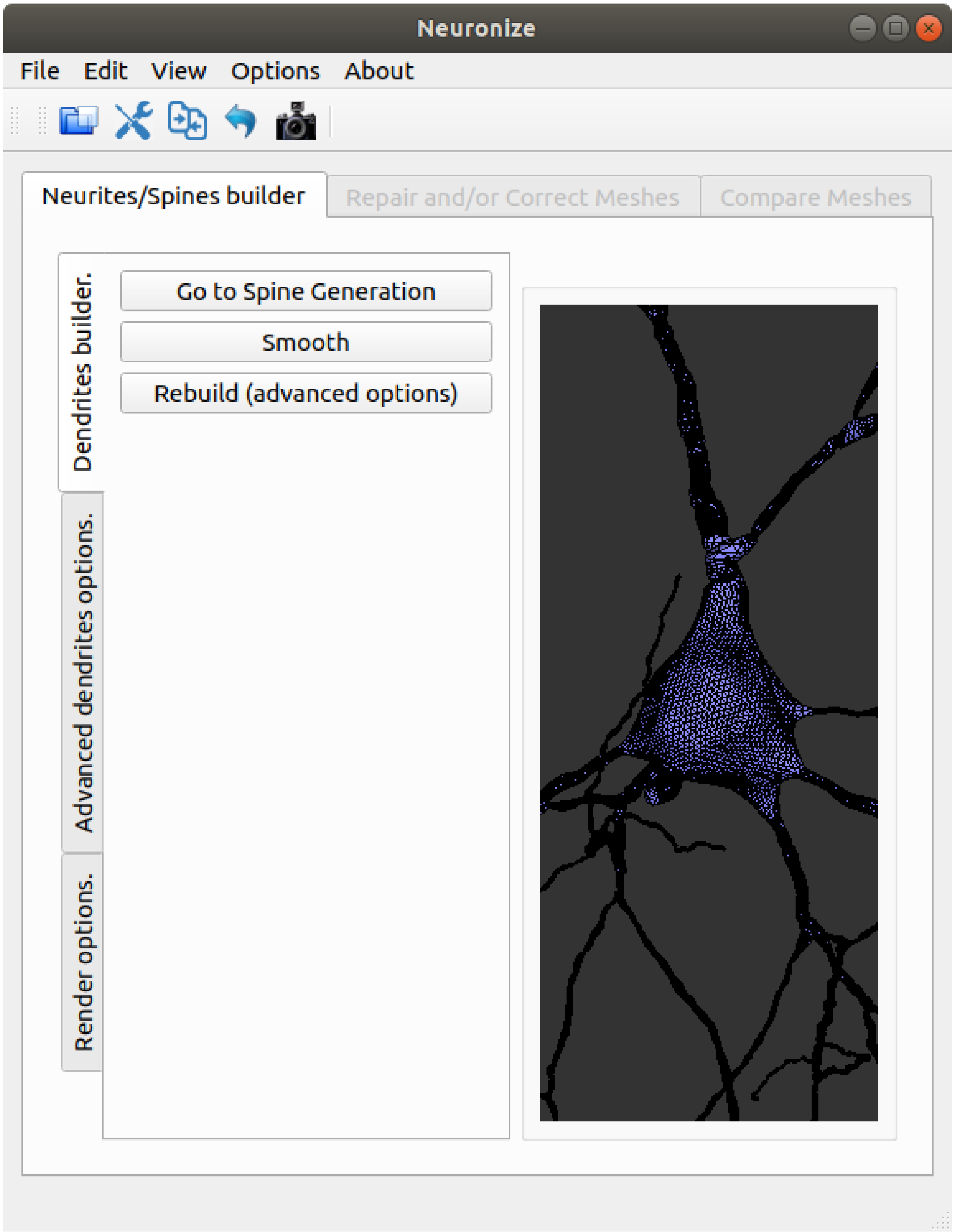

Figure 7. Interface inside the stage of "Neurites Builder" to generate the neurites.

At this point, the tool shows a dialog to select the desired spines to add (see Figure 8), being able to choose between:

- Spines included in this file: Load the spines contained in the VRML file.
- Spines from default dataset: Load the geometry of the spines of a local database.
- Spines from a new file: load spine meshes from other file (i.e. Imaris isosurface spines). Note that the loaded meshes must be at the same spatial coordinates as the loaded neuron.

Keep in mind that some options are not available for some input data.

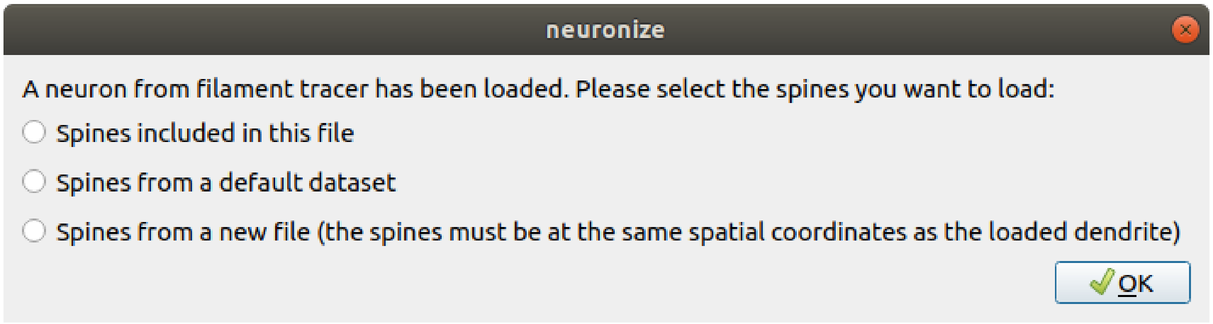

Figure 8. Dialog that allows the user to select the desired type of spines geometry to add to the current neuron.

When the process to add the spines is over, the user can examine the neuron with the added spines. Once finished the exploration, the user can start again all the generation process by pressing the "Go Home" button.

2.2 Generation a set of neurons.

To automatically generate a set of neurons, select the "Set of Neurons" button (see Figure 1). Then, a dialog is opened (see Figure 9) to select the input directory, which must meet the following conditions (please, note that example names are shown in italics for clarification purposes):

1. The main folder, e.g. folderSeveralTracings, should contain other folders with Imaris filament Tracer Files or tracing files.
    a. The tracing files must be in the root of the folder and their name will be considered the name of the neuron e.g. folderSeveralTracings/neuron1.asc or folderSeveralTracings/neuron.swc
    b. The Imaris Filament Tracer files must be in an independent folder, and the name of this folder must be the name of the neuron. e.g. folderSeveralTracings/Neuron1.
        i. If the neuron has an apical dendrite, this folder may contain a file with all the information of the apical dendrite. The file name must contain the text "api". e.g.folderSeveralTracings/Neuron1/TracingFileApi.vrml
        ii. Also, this folder must contain all the files related to the basal dendrites, and these files must contain the text "basal" in their names. These files can be directly in the folder of that neuron or insubfolders e.g. folderSeveralTracings/Neuron1/FichTrazadoBasal.vrml and folderSeveralTracings/Neuron1/BasalDendrites/basal1.vrml.
        iii. Besides, if the neuron has an Imaris generated soma, the folder may contain a file with the soma information. The file name must contain the text "soma", e.g. folderSeveralTracings/Neuron1/NeuronNameSoma.vrml

iv. The tool also allows adding the Imaris surface generated spines, by adding a new file that contains this information. This file name must contain the text "vols".
e.g. folderSeveralTracings/Neuron1/spinesVols.vrml
v. If the provided Imaris Filament tracer files do not contain information about the spines (regardless of whether Imaris Surface generated spines are provided), its possible to add an Imaris Longs file (containing the lengths of the spines) in order to generate a tracing with spines. The file name must contain the text "longs"
e.g. folderSeveralTracings/Neuron1/spinesLongs.vrml

Note that the tool does not allow to select a specific type of spine to be added, since the tool automatically selects the type of spine that provides more accuracy in function of the provided input files (the order from less to more accuracy is: "Distributed spines", "Real positions of ASC", "Real position and Geometry filament tracer" and "Real position and orientation Imaris)

Next, the output directory is selected. When the process has finished, for each processed neuron, a folder will be generated. These folders contain the tracing of the neuron, the neuron mesh in OBJ format, the spines meshes and the soma mesh in the same format. Note that, when generating tracings from Imaris Filament Tracer files, each generated tracing file will be available in its corresponding output folder, allowing the unsupervised automatic generation of multiple tracings.

Finally, the tool has two configuration parameters:

- ***Subdivisions***: this parameter controls the number of times that a smoothing process is applied to the neuron.
- ***Base Name***: this parameter allows selecting the beginning of the name of the folders that store the information generated by the tool for each neuron.

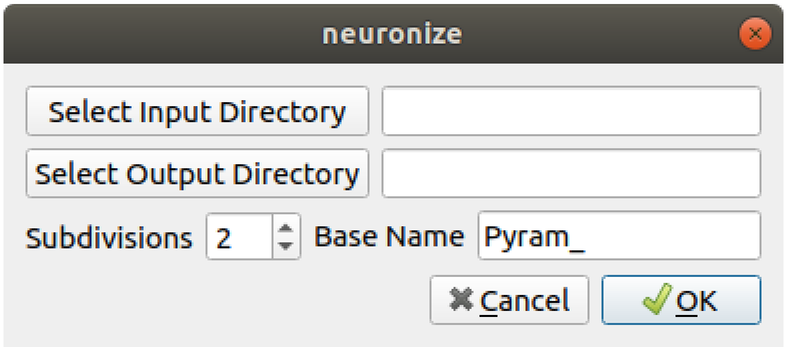

Figure 9. Interface to generate multiple neurons in an unsupervised way. The input directory must have a specific structure for the automatic generation.

3 Mesh Repair

To repair meshes, the user clicks on the "Repair and/or Correct Meshes" tab (see Figure 10). In this tab, the user can choose between repairing a single input file, or performing the repairing process on a set of input files.

If the user wants to repair only one file, the "File" button must be selected. Next, the user selects the input file (VRML or IMX format) and introduces the output file (CSV format) to store the metrics calculated from the repaired meshes.

If the user wants to repair a set of files at once, he/she selects the "Folder" button. Next, the input folder (that contains all the files to be processed) is selected, and the name of the output folder where the calculated metrics will be stored is inserted.

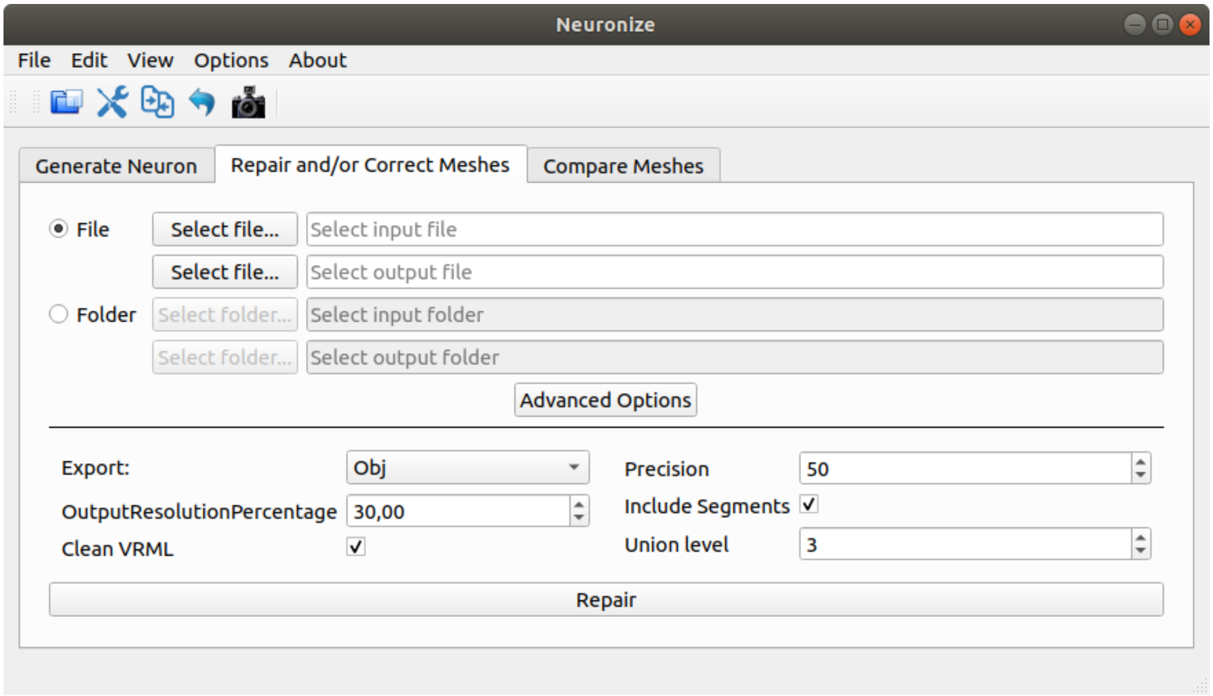

Figure 10. Interface to repair meshes.

If the user clicks on "Advanced Options", an advanced view allowing the configuration of some parameters of the repair process is displayed. Note that, by default, the parameters are configured to repair dendritic spines.

These advanced configuration parameters are:

- ***Export***: this parameter allows the user to select the output format of exported meshes. The exported meshes are stored in the same directory of the output file (or in the output folder in the case of multiple files) inside of a folder named "Meshes", in a folder with the same name as the input file. e.g. outputFolder/Meshes/InputFileName.
- ***OutputResolutionPercentage***: The repaired meshes are calculated at high resolution for an accurate calculation of the metrics. However, the user usually wants to store the mesh with a lower resolution (to use less disk space). This parameter allows controlling the reduction of the quality of the exported meshes.
- ***Clean VRML***: This parameter controls if the algorithm performs a pre-process of the input VRML file to delete the parts that are not useful. This parameter should be disabled if the input file does not come from Imaris or Imaris Filament Tracer.
- ***Precision***: this parameter controls the accuracy of the algorithm when repairing the meshes. Higher values get more accurate results with respect to the input meshes. However, a too high value could saturate the memory use causing the application to end.

- ***Include Segments***: this parameter only applies if the input files come from Imaris. Such files can contain information from spines, and fragments or neuritic segments. This parameter is activated when the user wants to repair not only the spines, but also the neuritic segments.
- ***Union Level***: this parameter controls the aggressiveness of the algorithm when merging different unconnected parts of the meshes. In addition, it also controls the degree of smoothing applied to the mesh.

Note that, when passing the cursor over the parameters names, a small help box, explaining the function of each of them, will be displayed.

3.1 Installation and use requeriments

In order for this application module to be available, it is necessary to have *Python3* installed on the system. If the user wants to repair meshes and does not have *Python3*, the user must first install it from the official python site (https://www.python.org/ftp/python/3.7.4/python-3.7.4-amd64.exe).

If the operating system is Ubuntu, in addition to *Python3* it is also necessary to have *virtualenv* installed. For that the user must do:

```
sudo apt-get install python-virtualenv
```

4 Comparison between two meshes

To compare meshes, the first step is to go to the “Compare Meshes” tab (see Figure 11). In this tab, users must select the meshes that they want to view in the section of “Meshes to be viewed” (the tool support OBJ, OFF, PLY and STL formats).

To compare two meshes, users should check the “Meshes to be compared” option and select both meshes. Once the meshes are selected, the tool shows the meshes colored according to the distance between them (warm colors indicate greater distances). Note that, it is possible that the selected meshes are not in the same position, and therefore the distances between them are very high. If this happens, the tool shows a small dialog (see Figure 12) that offers the user to continue with the meshes in their original position, or instead, to center both meshes and measure their distances again. Note that the two options for viewing and comparing meshes are compatible and can be used at the same time, allowing comparing two specific meshes (e.g. two spines) while visualizing them within their context (e.g. their dendrites and other spines).

This tab allows moving the camera in the visualization area to more effectively explore the differences found. In addition, the camera movements are synchronized between both visualizations, so that a camera movement is observed in each of them.

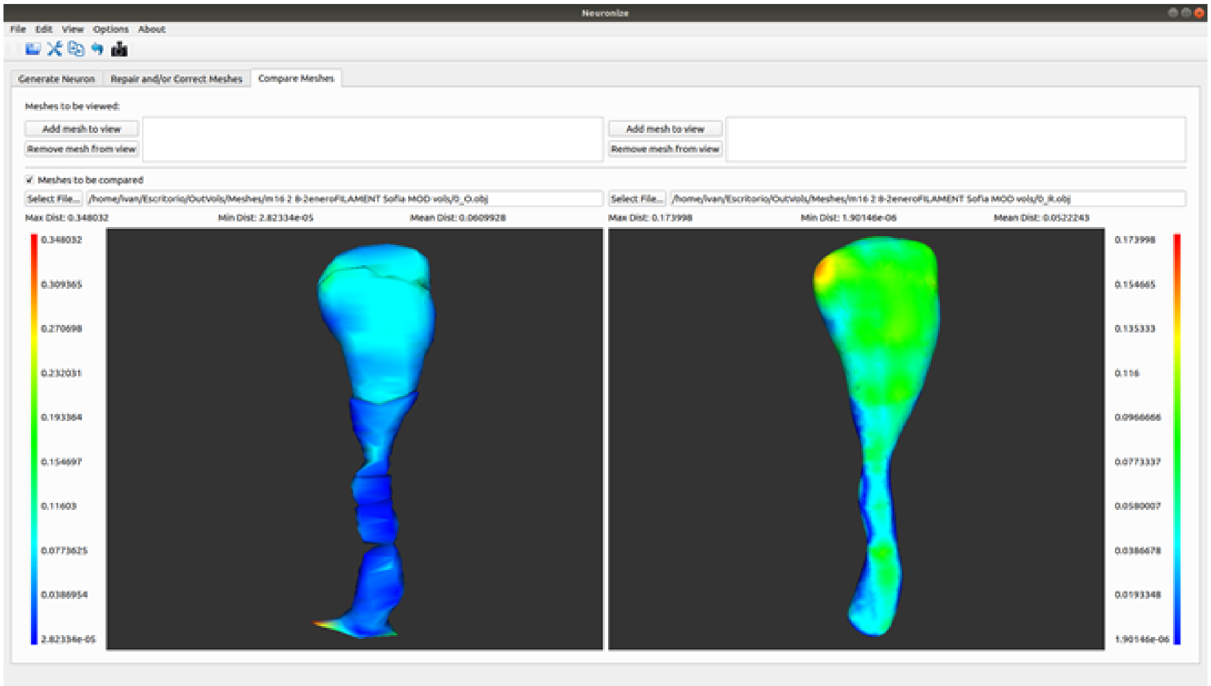

Figure 11. Interface showing the comparison between the two meshes.

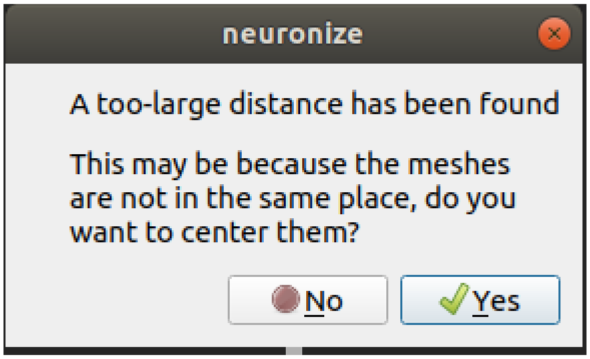

Figure 12. The dialog shows that the distance found between the meshes is very high.

5 Export information from the database

Finally, the tool has a small local database that stores all the neurons generated by the tool, and all the repaired spines.

The tool allows the user to export metrics related to the soma and spines to CSV files for each neuron contained in the database. To do this, the user must click on the menu “File” -> “Export Neuron Info” (see Figure 13).

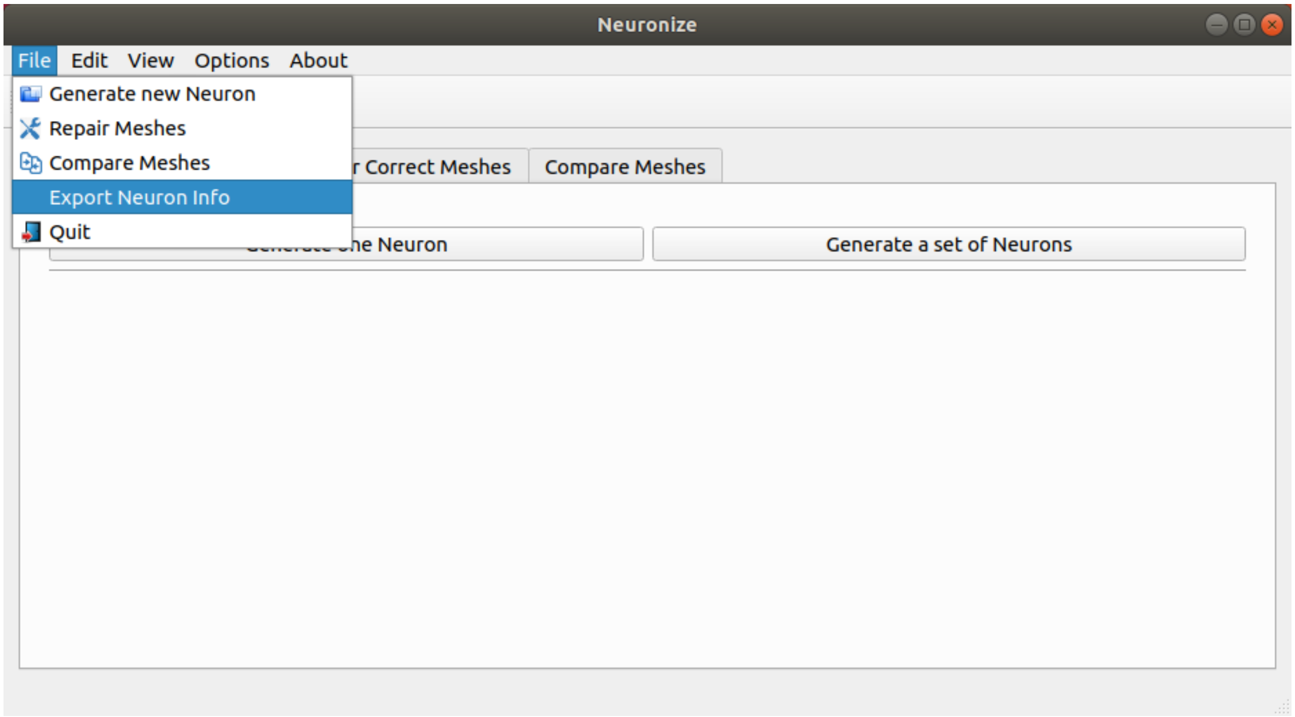

Figure 13. Menu to export neurons to a database.

Next, a dialog is opened (see Figure 14). In this dialog, the user selects the neurons to be exported and clicks on the "OK" button. Then, the user selects the path to store the exported CSV files. Once the files are exported, there will be one folder for each neuron that will contain two CSV files, one containing the soma information and another containing the spine information.

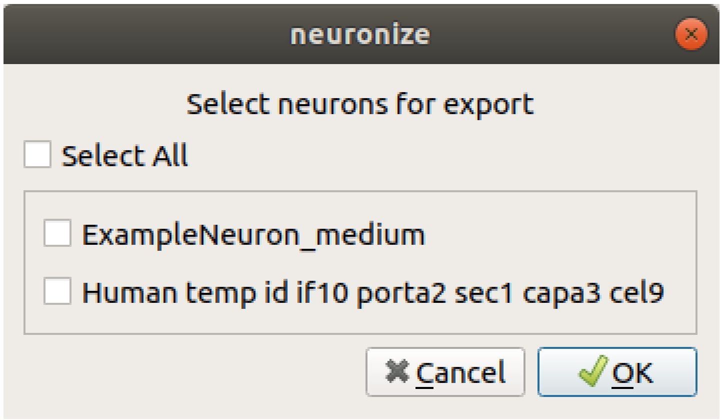

Figure 14. The dialog that allows selecting the neurons to export from the database to CSV files.
